# Imaging Spectrum of HTLV-1–Related Neurologic Disease: A Pooled Series and Review

**DOI:** 10.1212/CPJ.0000000000200147

**Published:** 2023-03-27

**Authors:** Luke Dixon, Cillian McNamara, Divya Dhasmana, Graham P. Taylor, Nicholas Davies

**Affiliations:** Department of Neuroradiology (LD, CM), Imperial College Healthcare NHS Trust, London, UK; National Centre for Human Retrovirology (DD, GPT, ND), Imperial College Healthcare NHS Trust, London, UK; Section of Virology, Department of Infectious Disease (GPT), Imperial College London, UK; Department of Neurology (GPT), Imperial College Healthcare NHS Trust, London, UK; Department of Neurology (GPT), Chelsea and Westminster Hospital NHS Trust, London, UK.

## Abstract

**Purpose of Review:**

Human T-cell lymphotropic virus type 1 (HTLV-1)–associated myelopathy (HAM) is a well-recognized neurologic complication of HTLV-1. Beyond HAM, several other neurologic manifestations are increasingly recognized, including acute myelopathy, encephalopathy, and myositis. The clinical and imaging features of these presentations are less well understood and potentially underdiagnosed. In this study, we summarize the imaging features of HTLV-1–related neurologic disease, providing both a pictorial review and pooled series of the less well-recognized presentations.

**Recent Findings:**

35 cases of acute/subacute HAM and 12 cases of HTLV-1–related encephalopathy were found. In subacute HAM, cervical and upper thoracic longitudinally extensive tranverse myelitis was noted, while in HTLV-1–related encephalopathy, confluent lesions in the frontoparietal white matter and along the corticospinal tracts were the most prevalent finding.

**Summary:**

There are varied clinical and imaging presentations of HTLV-1–related neurologic disease. Recognition of these features aids early diagnosis where therapy may have the greatest benefit.

In the early 1980s, the first human retrovirus was described—human T-cell lymphotropic virus type-1 (HTLV-1).^1^ Three related viruses were subsequently recognized: HTLV-2, HTLV-3, and HTLV-4. So far, only HTLV-1 is securely linked to human disease.^2^ It is estimated that 10 million people are infected worldwide with HTLV-1. It is endemic in diverse geographical regions, including southwestern Japan, equatorial Africa, and South America.^3^ The virus is transmitted through breastfeeding, sexual intercourse, direct blood contact, and organ transplantation.^4,5^ Infection is lifelong, but most persons living with it are asymptomatic. The most feared complications are HTLV-1–associated myelopathy (HAM), also known as tropical spastic paraparesis (TSP) and adult T-cell leukemia/lymphoma (ATL), the lifetime risk of each being 3–5%. Recently, there has been renewed attention to the broader spectrum of potential HTLV-1–related CNS complications and an increased focus on the prevention and improved treatment of HTLV-1–related disease.^6^ This paradigm shift highlights the need for better awareness of the potential imaging presentations of HLTV-1 and for more robust systematic approaches to imaging and follow-up. This review focuses on the documented non-ATL neurologic associations, including HAM, acute myelopathy, and HTLV-1–related cerebral disease.^7^ To inform this review, we have pooled our own and published cases for the less-characterized acute myelopathy and encephalopathy presentations.

## Search Strategy

After local institutional board review, a retrospective case note review was performed on all patients with HTLV-1 presenting to the National Center for Human Retrovirology, Imperial College Healthcare NHS Trust, London. Routinely acquired retrospective data were obtained and anonymized before analysis, and informed patient consent was deemed not required.

Patients who presented with acute to subacute myelopathy or encephalopathy were pooled with previously published cases, which were identified through searching MEDLINE and EMBASE using Ovid online. The search date range was from inception to week 1 of 2022, and the search enquiry used the following keywords “HTLV” OR “HTLV-1” OR “HTLV-I″ AND “neurologic” OR “neuro*” OR “brain” OR “cerebral” OR “spine” OR “myelopathy” OR “HAM” OR “TSP” OR “paraparesis” OR “encephalopathy” OR “encephalitis” OR “enceph*.” Cases where there was another potential cause of myelopathy or encephalopathy were excluded. Please see supplementary material for a detailed summary of the pooled cases.

## Pathophysiology

HTLV-1 chronically infects T lymphocytes, where it is predominantly found in a proviral form. Most patients remain asymptomatic, with the host's T-cell response primarily dictating the risk of secondary disease. HTLV-1 indirectly damages the CNS through infecting CD4^+^ T cells, which cross the blood-brain barrier and activate cytotoxic CD8^+^ T cells causing neuroglial death and degeneration.^8,9^ A high proviral load (PVL) and a PVL greater in the CSF than in the blood are risk factors of developing HAM.^10^ The PVL and the host's immune response are affected by factors such as the host's human leukocyte antigen genotype and single-nucleotide polymorphisms.^11^ The CSF concentrations of the cytokines neopterin and CXCL10 (IP-10) correlate with the rate of disease progression in HAM. CXCL10 also drives chronic inflammation by stimulating the migration of infected T cells into the CNS.^12^ The transmission route also seems to be a determinant factor for different potential complications. ATL has a stronger association with infection during infancy, predominantly through mother-to-child transmission, while HAM is associated with later infection, predominantly through sexual transmission or infected blood transfusions.^13^

## Diagnosis of HTLV-1 Infection

Screening tests detect the presence of antibodies to HTLV-1 or HTLV-2 in sera. Reactive samples undergo confirmatory testing using a Western blot or line immunoassay to type the infection. Molecular testing by PCR can also confirm and type HTLV infection but additionally can provide quantitative evidence of HTLV-1 or HTLV-2 and is mainly reported as a percentage of peripheral blood mononuclear cells infected. PVL less than 1% is considered low and typically unassociated with disease from HTLV compared with that more than 1%. Further risk stratification of asymptomatic carriers into those at an increased risk of myelopathy is aided by measuring markers of T-cell activation, i.e., cell surface markers on CD4 and CD8 cells.^14^

### HTLV-1–Associated Myelopathy

HAM is a neuroinflammatory disease of the spinal cord. Patients with HAM typically experience chronic lower back pain with early neuropathic urinary bladder symptoms followed by the development of lower limb spasticity.^15^ Proximal weakness of the lower limbs, which eventually spreads distally, is common.^16^ The onset of HAM is variable, although the chronic, slowly progressive presentation is most characterized. Acute and subacute presentations, with progression to severe paraplegia in only a few months, can occur.^17^ Poorer prognostic indicators in patients with HAM include a high HTLV-1 PVL, female sex, age at onset of 50 years or older, and an early rapidity of progression.^18^

### Chronic HTLV-1–Associated Myelopathy

Chronic HAM is diagnosed based on characteristic clinical features and confirming the presence of HTLV-1 infection. The WHO proposed diagnostic criteria for HAM in 1989 have since been modified by De Castro-Costa et al. to separate the degree of certainty of diagnosis as definite, probable, or possible (eTable 1, links.lww.com/CPJ/A405).

**Figure 1:**
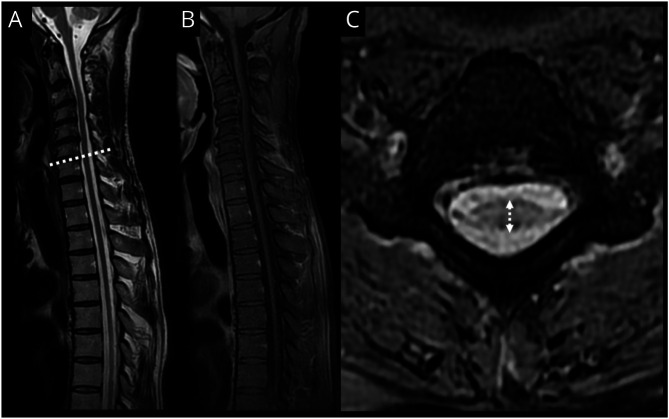
Spinal MRI in Chronic HAM Sagittal T2w and postcontrast, fat-saturated T1w (A and B) and axial T2w (C) MR images at the level of the cervicothoracic junction in a patient with chronic HAM demonstrates long-segment diffuse spinal cord volume loss greatest in the anteroposterior dimension.

In chronic HAM, progressive inflammation results in extensive white matter degeneration over time^19^; preferentially affecting the thoracic cord resulting in cord atrophy with predominant lateral and central posterior column involvement.^20^ Eventually, axonal loss extends to involve most of the spinal cord.^21^ On MRI, the cross-sectional area of the spinal cord is smaller in those with chronic HAM compared with that in healthy controls, and the degree of volume loss correlates with an expanded disability status scale.^22^ Diffuse cervicothoracic cord atrophy is most common, followed by thoracic cord atrophy only (Table 1). In the rapidly progressive form, the rate of spinal cord volume loss is approximately 5–10% per year.^22^ Volume loss is predominantly in the anteroposterior dimensions (Figure 1), with histopathologic assessment demonstrating preferential atrophy in the white matter of the lateral columns.^23^ This has been postulated to be explained by decreased glucose metabolism in this region secondary to reduced relative perfusion.^24^

**Table 1:**
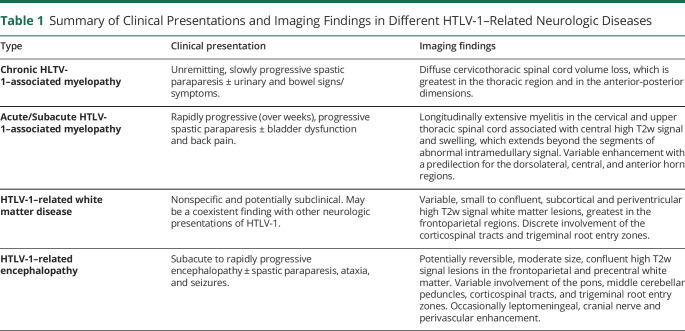
Summary of Clinical Presentations and Imaging Findings in Different HTLV-1–Related Neurologic Diseases

### Acute and Subacute HTLV-1 Myelopathy

Although HAM has historically been viewed as a chronic unremitting disease, it can have a more acute or subacute onset, which can wax and wane in severity. Awareness of this potentially under-recognized presentation is important because preliminary evidence suggests benefit from early immunosuppressive treatment.^6^ The prevalence of subacute forms of HAM ranges between 5 and 20% in different series.^25^ A pooled review (eTable 2, links.lww.com/CPJ/A405) of our own (n = 6) and published cases (n = 29) found 35 cases of HTLV-1–related acute/subacute myelopathy. In most cases (91%), there was no history of blood transfusion or organ transplantation, suggesting sexual contact may be the predominant transmission route. Of the cases where gender was reported, 76% were female, which mirrors the observation of a worse prognosis in female patients with chronic HAM. Of the cases where CSF PMBC HTLV-1 proviral load were performed, in most patients, it was higher in the CSF than in the peripheral blood. Where a trial of steroid therapy was given, 88% reported a noticeable improvement in clinical symptoms and radiologic appearances.

**Figure 2:**
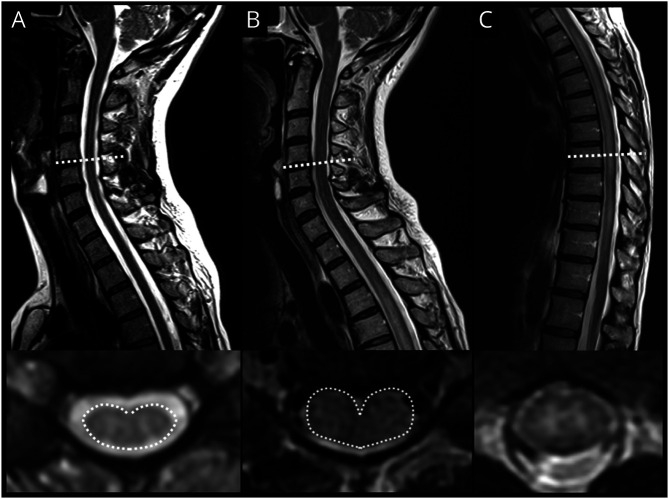
Spinal MRI in Subacute Rapidly Progressive HAM With Severe Spinal Cord Swelling Sagittal (top) and axial (bottom) T2w images 10 years before the presentation (A) and at presentation (B and C) in a 55-year-old woman with a 2-month history of rapidly progressive spastic paraplegia and bladder dysfunction. Imaging at nadir (B and C) demonstrates a new long-segment high T2w signal in the central thoracic spinal cord and diffuse swelling, which involves the entire spinal cord extending beyond the region of abnormal signal with a “heart symbol”–like appearance to the cervical spinal cord (B, bottom image).

Where MR imaging was performed (n = 16), the most frequently observed pattern was acute longitudinally extensive transverse myelitis in the cervical and upper thoracic spinal cord (n = 13) with central high T2w spinal cord signal and swelling (Table 1). In all 6 of our local cases, we noticed that the diffuse swelling extended far beyond the region of high T2w signal, and in 2, the main finding was swelling without appreciable signal change. A potential qualitative sign of swelling is increased convexity to the ventral spinal cord on either side of the anterior median fissure, which gives an almost “heart symbol”–like appearance (Figure 2).

**Figure 3:**
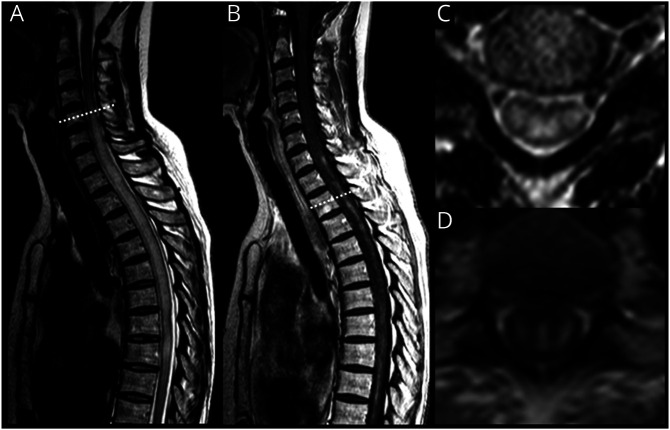
Spinal MRI in Subacute Rapidly Progressive HAM Demonstrating Enhancement A 54-year-old woman with subacute HAM presenting with a 1-month history of rapidly progressive paraplegia and bladder dysfunction. Sagittal T2w and contrast-enhanced T1w MR images (A and B) of the cervicothoracic cord shows longitudinal intramedullary hyperintensity and associated longitudinal enhancement. Axial T2w image at the upper thoracic cord (C) level shows high T2w signal and swelling involving gray and white matter but most severe in the central cord. Axial contrast-enhanced T1W (D) shows central and peripheral enhancement in the region of the lateral corticospinal tracts, which mimics the “trident sign” seen in neurosarcoidosis.

The presence and pattern of contrast enhancement in acute HAM are variable, with both dorsolateral and anterior horn involvement noted. In 1 local case, the pattern of dorsolateral and central spinal cord enhancement mirrored the “trident sign” previously described in spinal neurosarcoidosis (Figure 3).^26^ This enhancement pattern correlates with the histologic observation of lateral and central dorsal column predominant involvement.^27^ The pathologic observation of spinal white matter tract vacuolation may partly account for the observed radiologic finding of spinal cord swelling.^27^

## Differentiating HAM From Neuromyelitis Optica

Differentiating acute HAM from neuromyelitis optica (NMO) can be challenging, especially in countries with a high prevalence of both, such as in Brazil and Japan.^28,29^ While ocular manifestations of HTLV-1 such as uveitis and keratoconjunctivitis are well recognized, there are only minimal reports of HTLV-1–related optic neuritis, suggesting this is more a feature of NMO.^30^ Current evidence indicates that aquaporin-4 antibodies are not directly related to HTLV-1–associated myelopathy, neither in the pathogenesis of HAM nor in the development of AQP4 antibodies post–HTLV-1 infection.^31^ In endemic areas, testing for both HTLV-1 and AQP4 antibodies is therefore recommended. Additional clinical features suggestive of HAM over NMO include older median age at onset and lower EDSS scores with a generally slower progressive course.

### Cerebral HTLV-1

Awareness of the clinical and radiologic manifestations of cerebral HTLV-1 is increasing, with chronic white matter lesions and acute encephalopathic presentations now recognized. Patients with HAM have more significant neurocognitive deficits than asymptomatic carriers and age-matched controls. Advanced imaging studies support the presence of brain inflammation in HTLV-1.^11^C-PBR28, a PET tracer that binds to translocator protein (TSPO) on microglia and is used as a marker of neuroinflammation, showed increased cerebral, thalamic, and brainstem TPSO binding in patients with HAM with severe disease compared with those with milder disease.^32^

### White Matter Lesions

**Figure 4:**
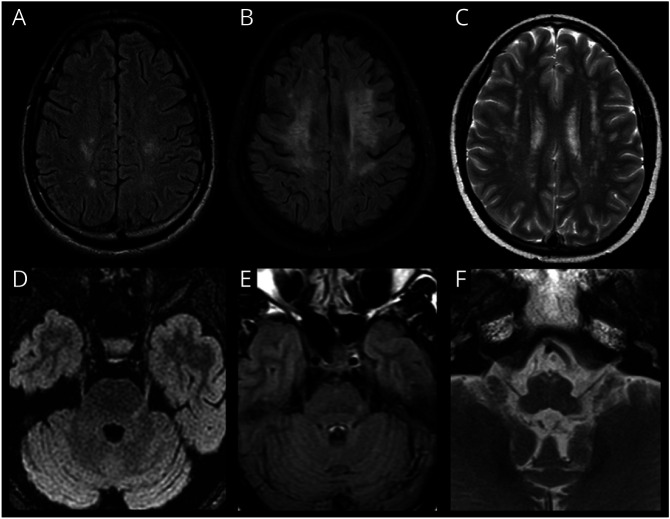
MR Imaging in HTLV-1–Related White Matter Disease Axial FLAIR (A and B) images of two different patients with HTLV-1 demonstrated symmetrical lesions in the precentral white matter with the second patient (B) demonstrating more extensive confluent lesions in the frontal white matter. Axial T2w image (C) of a 24-year-old woman with chronic HAM since 12 years of age demonstrates multiple partially confluent high T2w lesions in the deep and subcortical frontoparietal white matter. Axial FLAIR images of two different patients with HAM (D and E) both demonstrating lesions along the left trigeminal root entry zone. Axial T2w image of a patient with HAM and encephalopathy (F) demonstrating symmetrical lesions in the medullary pyramids along the path of the corticospinal tracts.

Patients with HAM have a more significant number of cerebral white matter (WM) lesions than age-matched controls on MRI and postmortem studies.^33^ The lesions are believed to be secondary to gliosis from chronic perivascular inflammation. There is a positive correlation between the extent of cerebral white matter lesions and the degree of spinal cord involvement, suggesting synchronous cerebral and spinal disease.^34^ The clinical significance of WM lesions is uncertain, although a correlation between lesion burden and verbal memory deficits has been noted.^35^ The WM lesions vary from small to extensive and confluent with typically ill-defined boundaries (Table 1). They are predominantly noted in the subcortical and periventricular regions, with a predilection for the bilateral frontoparietal white matter and the ventral centrum semiovale (Figure 4).^35^ In our group of 6 patients with rapidly progressive CNS disease, intracranial imaging has revealed a pattern of relatively symmetrical lesions along the path of the corticospinal tracts (n = 4) with widespread involvement of the median precentral white matter (Figures 3 and 4). Involvement of the cerebral pyramidal tracts is observed in other published cases.^36^ Chronic linear lesions along the trigeminal root entry zones were also observed in two-thirds of patients in our same cohort (Figure 4). These mimic the lesions seen in multiple sclerosis (MS), although, unlike MS, there are no corresponding trigeminal symptoms. However, in endemic areas, cranial nerve palsies have been reported in association with HTLV-1.^37^ In 1 case of presumptive HTLV-1–related encephalopathy, bilateral trigeminal nerve enhancement has also been noted (see HTLV-1–related encephalopathy).^38^

### Multiple Sclerosis vs Cerebral HTLV-1

Discriminating the white matter lesions of HTLV-1 from multiple sclerosis (MS) is potentially problematic. In a small series comparing 12 patients with HAM with 13 patients with MS, the combination of a white matter lesion >6 mm and a periventricular lesion >3 mm was found to achieve the highest specificity and sensitivity for MS.^39^ Brain stem and cervical spine lesions are also purported to be more characteristic of MS.^40^ In a Japanese cohort, the Fazekas criteria demonstrated reasonable accuracy for differentiating MS from cerebral HTLV-1 (93% specificity, 86% sensitivity).^41^ Other clinical and laboratory factors aid differentiation of MS from HAM. Primary progressive MS can mimic the caudal rostral pattern of HAM but optic nerve involvement common in MS is rare in HAM. Intrathecal production of oligoclonal IgG bands is found in both conditions, but as described earlier, HTLV-1 proviral measurements in the blood and CSF help identify those with high HTLV-1–related inflammation and CNS disease, respectively.

### HTLV-1–Related Encephalopathy

There is increasing evidence that HTLV-1 can be associated with encephalopathy. A prior case series published by our group reported 3 local cases and pooled them with 6 previously published cases.^42^ In this series, the annual incidence of encephalitis in the UK national HTLV-1 cohort (n = 142) was approximately 50 times higher (rate of 278/100,000) than the reported annual incidence in the UK general population.^42^ We have since identified an additional Brazilian case and 2 further UK cases of otherwise unexplained encephalopathy in patients with HTLV-1 (2 reported and 1 unreported, eTable 3, links.lww.com/CPJ/A405).^38,43^ Of these 12 cases, most of them had preexisting HAM (83.3%), and all but 1 were female (92%).

**Figure 5:**
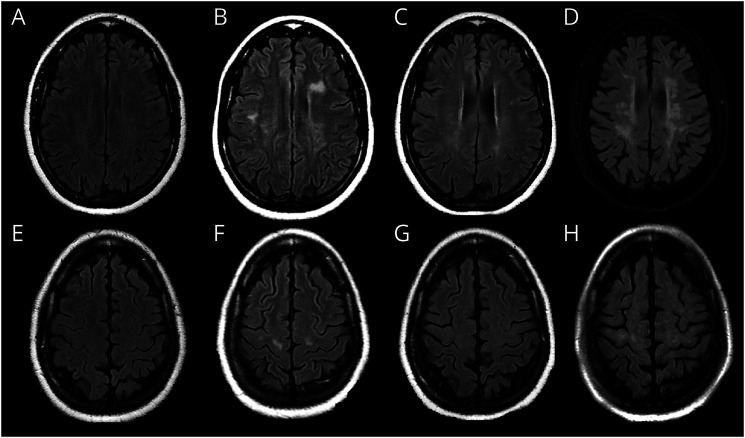
MR Imaging in a Patient With HTLV-1–Related Encephalopathy Serial axial FLAIR images at different time points over a 9-year period in a female patient with HAM and recurrent encephalopathic episodes. The first column (A and E) 9 years ago when the patient had established HAM demonstrates minimal subtle abnormal signal in the centrum semiovale. MRI 1 year later (B and F) shortly after an acute encephalopathic episode characterized by worsening upper limb weakness, diplopia, and ataxia demonstrates new bilateral lesions in the precentral white matter with additional deep and periventricular lesions in the frontal white matter. MRI 4 years later (C and G) demonstrates regression of the lesions with only subtle, Ill-defined residual abnormal signal. MRI 5 years later (D and H) during an episode of rapidly worsening HAM and impaired cognition demonstrates recurrence of the precentral lesions and new frontal white matter lesions.

**Figure 6:**
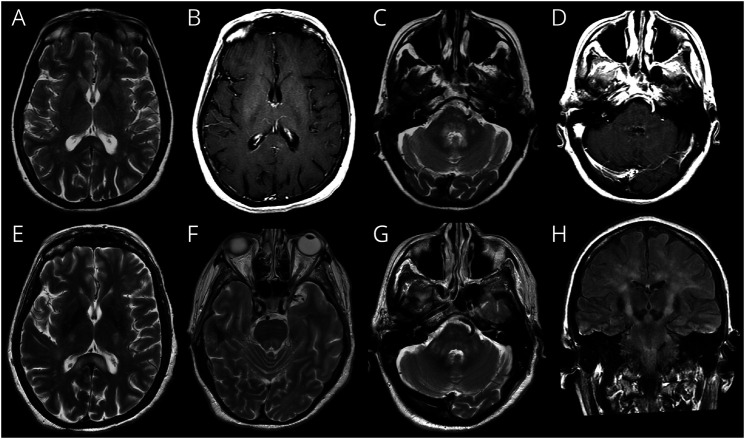
MR Imaging in a Patient With HAM and HTLV-1–Related Encephalopathy MRI of a female patient with HAM presenting with rapidly worsening weakness, headaches, a 6th nerve palsy, and a high CSF PVL of 120%. Axial T2w (A and C) and postcontrast T1w (B and D) images demonstrates a left splenial lesion that demonstrates faint enhancement (A and B) and further abnormal signal with subtle enhancement around the 4th ventricle in the dorsal pons, middle cerebellar peduncles, and dentate nuclei. After IV corticosteroids, the patients' symptoms improved and the lesions regressed (not shown). Eight months later, after debridement of a sacral decubitus ulcer, the patient had a new episode of deteriorating consciousness with recurrence of the 6th nerve palsy. Axial T2w (E,F,G) and coronal FLAIR (H) images showed recurrent and more extensive splenial (E), dorsal pons, and middle cerebellar peduncle (G) lesions and new extensive confluent abnormal signal along both corticospinal tracts (H) from the precentral white matter, into the internal capsules (E) and pons (G).

In cases with MR imaging characterization (n = 9), 7 patients had abnormal intracranial imaging (Table 1). The commonest observation was relatively symmetrical, confluent areas of high T2w signal in the frontoparietal white matter (n = 5) followed by an abnormal signal in the pons (n = 4). Three cases demonstrated symmetrical lesions in the precentral white matter. In 2 of these, there were transient episodes associated with more confluent abnormal signal along the corticospinal tracts from the precentral white matter to the brainstem (Figures 5 and 6). Two cases demonstrated lesions in both middle cerebellar peduncles and along the trigeminal nerve root entry zones. Two-thirds of patients treated with corticosteroids (n = 9) clinically improved, and the remaining one-third died (2 of pneumonia). In our cohort, 3 patients showed corresponding partial regression of the white matter lesions on follow-up MRI. All 3 patients had subsequent relapses associated with recurrent and new lesions (Figures 5 and 6). In two-thirds of patients, there was also pathologic intracranial enhancement during particularly fulminant episodes. One patient demonstrated periventricular enhancement in the pons and middle cerebellar peduncles and separately in a splenial lesion (Figure 5), while the other showed leptomeningeal enhancement involving several cranial nerves in addition to multiple foci of perivascular enhancement in the deep cerebral white matter.^38,44^ The observation of leptomeningeal involvement is further supported by a case-control study that found HTLV-1 had the highest prevalence of leptomeningeal enhancement across a range of chronic neuroinflammatory conditions in endemic tropical areas.^45^ Abnormal meningeal enhancement in the context of HTLV-1 should also raise the differential of leukemic infiltration.

### Posterior Fossa Presentations

Cerebellar syndromes are a rarer presentation of HTLV-1.^46,47^ Both chronic progressive cerebellar atrophy and an isolated case of acute cerebellitis with transient cerebellar swelling have been reported in the context of HTLV-1.^48,49^ Broken smooth pursuit eye movements and nystagmus have also been noted in patients with HAM, which may act as a marker for posterior fossa involvement.^50,51^

### Neuromuscular HTLV-1

**Figure 7:**
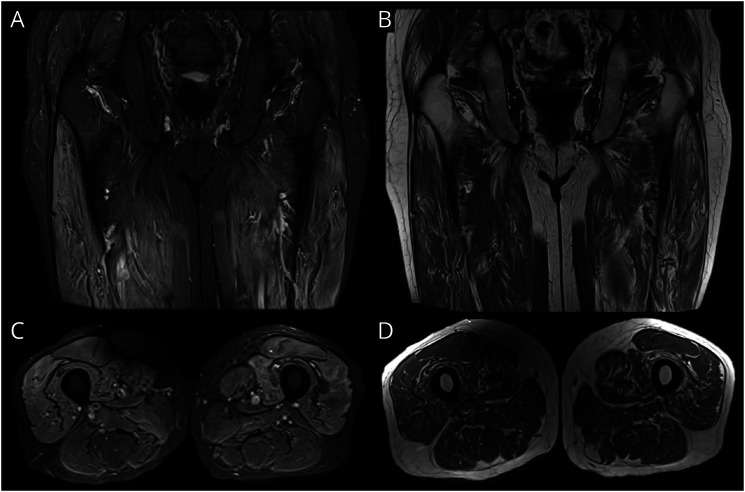
MR Imaging in a Patient With HTLV-1–Related Myositis Coronal and axial T2-weighted fat-saturated (A and C) and T1-weighted (B and D) images of a 70-year-old man with HTLV-1 and progressive myopathy over several years demonstrates edema and fatty infiltration into the anterior compartments of both thighs with relative sparing of the rectus femoris muscles.

Chronic HTLV-1 infection can be associated with motor neuropathy, and there have been cases mistaken for amyotrophic lateral sclerosis.^52^ In case-control studies of blood donors, polyneuropathy was described in 9% of seropositive individuals vs 3% in seronegative people.^53^ Electrodiagnostic studies have also shown that as many as 30% of patients with HAM may have concurrent polyneuropathy.^54^ Isolated HTLV-1 polyneuropathy without any spinal involvement tends to be predominantly sensory with distal paresthesia and stocking-type sensory loss.^7^ Inflammatory myositis has also been reported rarely in relation to HTLV-1, and HTLV-1 is myotoxic in vitro*.*^55^ The imaging features of HTLV-1–related myositis is poorly characterized, although in our cohort, we have seen a 70-year-old patient with HTLV-1 and an inclusion body myositis-like pattern where there was edema and fatty infiltration in the anterior compartments of both thighs, which relatively spared the rectus femoris (Figure 7).

### Treatment

There is currently no cure for HTLV-1–related neurologic diseases. Interferon α has shown some therapeutic benefit in a randomized control trial (RCT) in patients with HAM, but the efficacy is limited, and the treatment is rarely used due to potential risks.^56,57^ Corticosteroids are presently the mainstay of therapy and are used to suppress inflammation and improve or maintain long-term neurologic function. Evidence for corticosteroids is, however, limited and largely based on observational series and clinical experience.^58,59^ Choice of steroid therapy is based on the rapidity of onset and rate of progression. In rapid HAM progressors, high-dose induction with methylprednisolone followed by prednisolone maintenance is usually advocated, while in slow HAM progressors, low-dose prednisolone may be solely used.^60^ This stratification of presentation type and corticosteroid therapy has been supported by a recent RCT that, in both groups, demonstrated an improvement in motor disability scores and walking distance.^6^ Steroid-sparing, anti-inflammatory, disease-modifying maintenance treatment for HAM should be considered case by case where treatment with corticosteroids is not felt appropriate or where there is a need to wean off steroids due to complications. Beyond this, there is early evidence that anti-CCR4 monoclonal antibody therapy may have benefit, but this remains limited to clinical trials and is not readily available.^60^ Outside of immunomodulating therapy, the mainstay of management is supportive symptomatic relief and physiotherapy.

HTLV-1 is an underappreciated infection. Even in high-prevalence countries, there is limited awareness of its mode of transmission, disease course, and imaging features. The neuroradiologic manifestations of HTLV-1 go beyond spinal cord atrophy to include acute myelopathy, encephalopathy, and a wide range of associated imaging findings. Although data are too limited to reach definite conclusions, there are a few possible emerging imaging patterns that warrant additional investigation. The observation that immunotherapy may halt and, in some cases, reverse acute HTLV-1–related CNS disease further emphasizes the potential role of imaging in reaching an early diagnosis where therapy may be of greatest benefit. A recent WHO technical report described the pressing need for international collaborative studies to provide further insight into HTLV-1 pathogenesis and disease manifestation. Neuroimaging will likely be a key modality to achieving these aims.

**TAKE-HOME POINTS**

→ HTLV-related neurologic diseases are varied and extend beyond chronic HAM to include longitudinally extensive transverse myelitis, cerebral white matter disease, encephalopathy, and acute myositis.
→ HTLV-1 should be suspected and tested for in high-risk patient groups who demonstrate supportive imaging features (Table 1).
→ Acute HTLV-1–related neurologic diseases are potentially reversible, and early diagnosis may help prevent irreversible damage.
